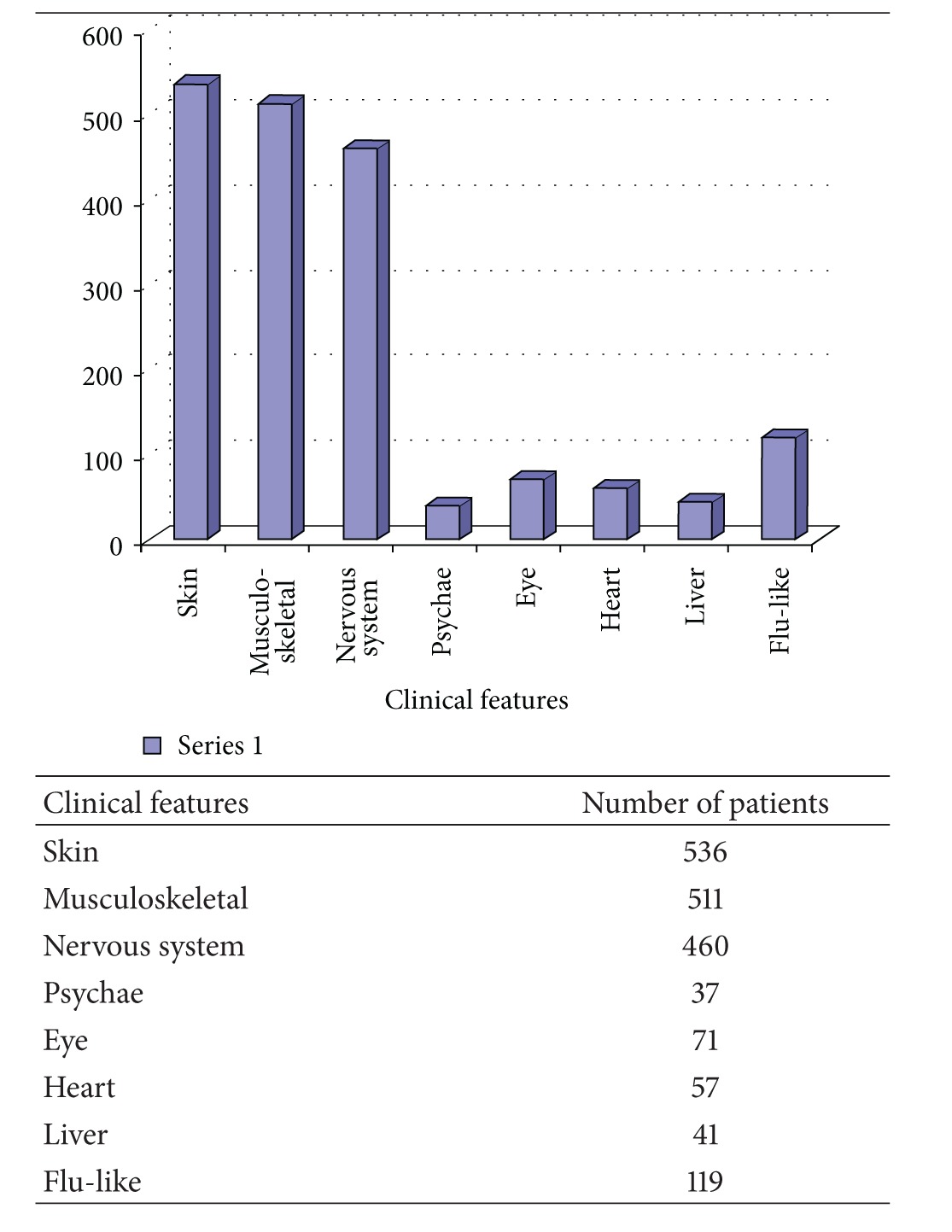# Clinical Features of 705 *Borrelia burgdorferi* Seropositive Patients in an Endemic Area of Northern Italy

**DOI:** 10.1155/2014/414505

**Published:** 2014-01-16

**Authors:** Giuseppe Stinco, Maurizio Ruscio, Serena Bergamo, Davide Trotter, Pasquale Patrone

**Affiliations:** ^1^Department of Clinical and Experimental Pathology and Medicine, Institute of Dermatology, University of Udine School of Medicin, Ospedale “San Michele,” Piazza Rodolone 1, Gemona del Friuli, 33013 Udine, Italy; ^2^Clinical Laboratory and Microbiology Department, Hospital of San Daniele del Friuli, San Daniele, Italy

## Abstract

*Background*. Lyme Borreliosis is a multisystemic infection caused by spirochetes of *Borrelia burgdorferi sensu lato* complex. The features of Lyme Borreliosis may differ in the various geographical areas, primarily between the manifestations found in America and those found in Europe and Asia. *Objective*. to describe the clinical features of Lyme Borreliosis in an endemic geographic area such as Friuli-Venezia Giulia in the Northeastern part of Italy. *Methods*. The medical records of patients resulted seropositive for *Borrelia burgdorferi* have been retrospectively recorded and analyzed. *Results*. Seven hundred and five patients met the inclusion criteria, 363 males and 342 females. Erythema migrans was the most common manifestation, detected in 437 patients. Other classical cutaneous manifestations included 58 cases of multiple erythema migrans, 7 lymphadenosis benigna cutis, and 18 acrodermatitis chronica atrophicans. The musculoskeletal system was involved in 511 patients. Four hundred and sixty patients presented a neurological involvement. Flu-like symptoms preceded or accompanied or were the only clinical feature in 119 patients. *Comments*. The manifestations of Lyme borreliosis recorded in this study are similar to the ones of other endemic areas in Europe, even if there are some peculiar features which are different from those reported in Northern Europe and in the USA.

## 1. Introduction

Lyme Borreliosis (LB) is a complex multisystemic infection that involves the skin, joints, nervous system, eyes, and heart, caused by spirochetes of the *Borrelia burgdorferi (Bb) sensu lato* complex, which are transmitted primarily by *Ixodid* ticks. After the transmission of the spirochete, human LB generally occurs in stages, with different clinical manifestations and evolution [1, 2]. The features of LB may be different in some aspects in the various geographical areas, primarily as regards the manifestations found in America and those found in Europe and Asia [3, 4]. The prevalence of erythema migrans (EM) is similar in the USA and Northern Europe, but lower in Southern Europe [3–5]. Acrodermatitis chronica atrophicans (ACA), borrelial lymphadenosis benigna cutis (LABC), and some manifestations of carditis have been detected almost exclusively in Europe [3–6]. Articular involvement seems to be more frequent in the USA than in Europe, where arthritis is more frequent in Southern countries rather than in the North [3]. Neurological damage is more often observed in European countries, whereas in North America milder forms were reported [3]. It may be supposed that even in Europe the main clinical features of LB can be different in some aspects. These assumptions prompted us to carry out a retrospective study aimed at assessing the clinical features of LB in an endemic geographic area as Friuli Venezia Giulia, in order to define and recognize the clinical presentations and the evolution of the disease in these lands and consequently to describe whether there are differences in the clinical presentations compared to those found in other areas of the world.

## 2. Materials and Methods

Every case history of each patient addressing to the regional reference centre for Lyme disease in San Daniele del Friuli from June 2004 to June 2010 has been retrospectively recorded and analysed. The medical records of patients that resulted seropositive for *Bb* and followed during the whole course of their disease were recruited. Patients who presented the following criteria have been excluded from the study: wrong diagnoses (not LB), seropositive patients who did not present any typical or occasionally associated manifestation of LB, seronegative patients, patients who did not report to the planned visits, subjects with EM without any other symptom who have been treated and cured and/or who made no serology test or seroconversion, and patients who made a prophylactic treatment after the tick bite or who were taking drugs that might have interfered with laboratory tests or could have hidden the clinical manifestations of LB.

Out of the 2000 analysed case histories, 705 met the inclusion criteria and were recruited in this survey. All of the subjects were resident in Friuli Venezia Giulia.

The ELISA (Enzyme-Linked Immunosorbent Assay) IgM/IgG and Western blot (IgM/IgG immunoblots if early disease was suspected; IgG Western blot alone if late disease was suspected) were performed to define seropositive patients, using the same kit in the same laboratory. Other laboratory and instrumental tests, such as tissue biopsy, culture, polymerase chain reaction (PCR), cerebrospinal fluid examination, sinovial liquid examination, haemochrome, liver function tests, ECG, X-rays, electromyography, and magnetic resonance, have been taken into account, particularly for the doubtful cases.

## 3. Results

Seven hundred and five medical records fulfilled the inclusion criteria, and thus were recorded and analysed in this study, including 363 males and 342 females, with a male to female ratio of 1.06. The mean age of the whole survey was 44.7 years, ranging from 4 to 86 years; 67 were under 16 years; 382 were between 16 and 60 years; 256 were over 60 years.

The clinical manifestations observed in the 705 patients seropositive for *Bb* have been summarized in Table 1.

There have been 536 (76.03%) cutaneous forms of LB recorded. EM was the most common manifestation and it has been detected in 437 patients (61.99%). There was neither gender difference (M : F = 215 : 222) nor age difference. EM has been found during the visit in 319 patients and it was reported by 118 subjects as an history fact. It appeared from 3 to 25 days after the tick bite (average: after 12 days). Other classical cutaneous manifestations of LB included 58 cases of multiple EM (8.23%), 7 LABC (1%), and 18 ACA (2.55%). Atypical cutaneous manifestations, that is, clinical features not surely linked to LB, were 16 (2.27%), in details: 8 cases of morphoea, 2 Raynaud's phenomenon, 1 lichen sclerosus and atrophicus, 1 pityriasis lichenoides, 1 Gilbert's pityriasis rosea, 1 urticaria, 1 anetoderma, and 1 erythema nodosum.

The musculoskeletal system was involved in 511 patients (72.48%): 262 out of 511 presented arthralgias or acute arthritis; they were 119 men and 143 women; 88 had a single episode whereas 174 suffered from several attacks. The evolution to chronic arthritis was seen in 22 cases (8% among the patients with arthralgias/acute arthritis). Muscular hypotonia and/or hypotrophy was observed in 41 patients (5.81%).

Four hundred and sixty patients presented a neurological involvement. Two patients reported Bannwarth's syndrome, 3 acute encephalopathy and 1 chronic encephalopathy. Two hundred and ninety-three patients (41.56%), 127 males and 166 females, reported cephalea, dizziness, memory and concentration problems, irritability, emotional lability, and sleepiness. As far as the peripheral nervous system is concerned, 161 patients (22.84%) reported paresthesias (115 cases), sensitiveness disorders (47 patients), and motion functionality disorders (19 cases). Out of these 161 patients, 74 suffered from neuritis, polyneuritis, meningoradiculitis and peripheral nerves paralysis, in particular there were 12 cases of the seventh cranial nerve paralysis.

Flu-like symptoms such as fever, arthromyalgia and headache preceded or accompanied or were the only clinical feature in 119 (16.88%) patients. Fever was the only symptom in 11 patients (1.56%) while in 165 (23.4%) cases was accompanied by other manifestations. A reactive regional or generalized lymphadenopathy was recorded in 75 patients (10.64%).

Psychiatric symptoms have been observed in 37 patients (5.25%), 22 men and 15 women. There were 21 cases of anxiety, 10 cases of depression and 6 complex syndromes characterised by panic attacks and phobias.

An intrinsic ocular disease or a disturb deriving from the neuromuscular involvement of the eye has been observed in 71 patients (10.07%), 25 males and 46 females: 28 cases of conjunctivitis, 7 cases of photophobia, 13 cases of visual impairment, 11 of bulbar pain, 4 of diplopia, 1 case of hemianopsia, 1 of orbital muscle tremor, 3 cases of lacrimation and burning and 3 of orbital oedema.

In 57 subjects (8.08%), 26 men and 31 women, there was an involvement of the cardio-vascular system: 14 patients suffered from tachycardia, 3 from bradycardia, and 13 from palpitations. Twenty-four patients presented with the acute onset of varying degrees of intermittent atrioventricular heart block, symptomatic or detected by the ECG. A case of complete atrioventricular block required the implant of a pacemaker. One patient referred angina and 2 patients suffered from a myocarditis.

An involvement of the liver was observed in 41 patients of this survey (5.81) who reported specific alterations of the cytonecrosis enzymes.

With the purpose of better investigating the severity spectrum of LB, an analysis of the summation of the number of symptoms per patient was made. Two hundred and forty-eight (35.1%) patients reported only 1 manifestation. Two different symptoms were observed in 251 (35.6%) patients, 3 clinical features were recorded in 120 (17.0%) subjects, and more than three clinical manifestations assignable to LB were reported for 86 (12.1%) cases.

## 4. Discussion 

Friuli Venezia Giulia is one of the endemic regions for LB in Italy because of the wide spread of piedmont zones which are full of underbrushes. Here the climatic, geographical, and faunistic conditions can foster the proliferation of the hard tick *Ixodes ricinus* and thus may represent an ideal ecosystem for the spread of *Bb* [7].

People living in Friuli Venezia Giulia may get in contact with the ticks over and over because the suspension of agricultural activities has caused the wide spread of woody areas.

In this study the seropositivity for *Bb* is seen in both males and females and it occurs at every age, even if the adults are more affected probably because they spend more time, for working or leisure reasons, in such zones with a thick vegetation. The observation that children are affected from a lesser extent than adults may be a result of the several campaigns of information for the prevention of LB which took place in recent years in our region. Therefore, the population has become very sensitive to this issue: people are accustomed to watching their children back from picnics or outdoor games in order to remove quickly the ticks.

Since the relatively high prevalence of antibodies against *Bb* (5% to 25%) is seen even in healthy persons, depending on their prior exposure to tick bites in their occupational and leisure-time activities [8], in our study, to avoid overdiagnoses, we excluded all seropositive subjects who did not present any other symptom related with LB.

The manifestations of LB recorded in this study are similar to the ones of other endemic areas in Europe, presenting a widespread clinical expressivity, even if there are some peculiar features which are different from those reported in Northern Europe and mostly in the USA, proving the existence of several genospecies of *Bb* differently distributed throughout the various geographical areas [4–10].

**Figure 1:**
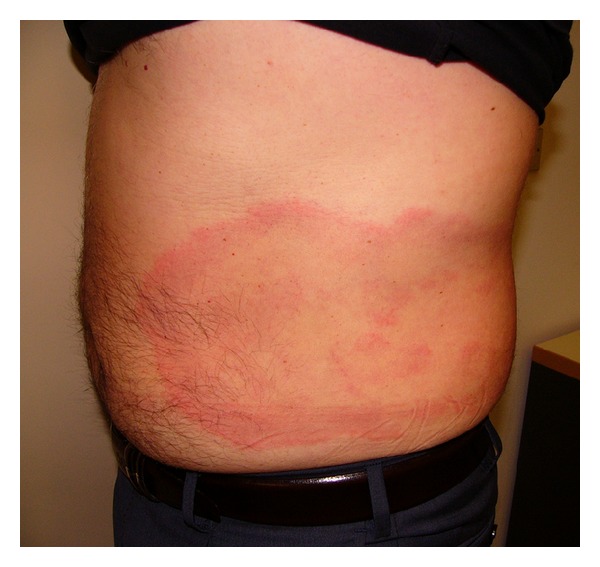
Erythema migrans.

A solitary EM lesion is the most frequent presentation of LB and EM was found to be the most common manifestation in the population analysed. Its clinical characteristics resulted similar to those reported in the Literature [2] (Figure 1). The lower incidence in this survey has been foreseen because of the exclusion of all the EM that has been treated and cured before the seroconversion.

**Figure 2:**
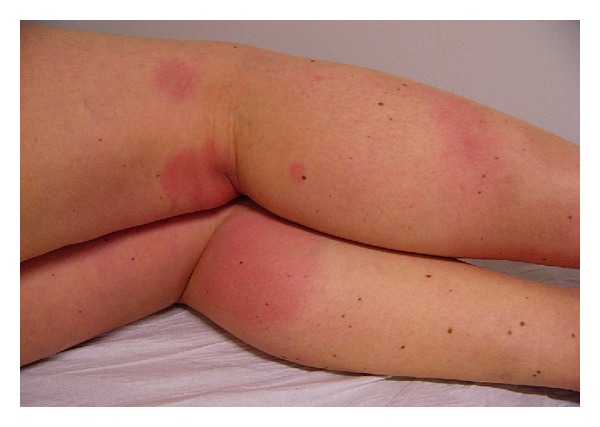
Multiple erythema.

In the USA, EM is often accompanied by flu-like symptoms such as fever, malaise, fatigue, headache, myalgia, or arthralgia, whether in Europe it more frequently represents an indolent and localised lesion [2, 10]. Multiple EM seem to be more often seen in USA (about 20%) than in Europe (about 10%) [11, 12]; in this study 58 cases (8.33%) have multiple EM (Figure 2).

**Figure 3:**
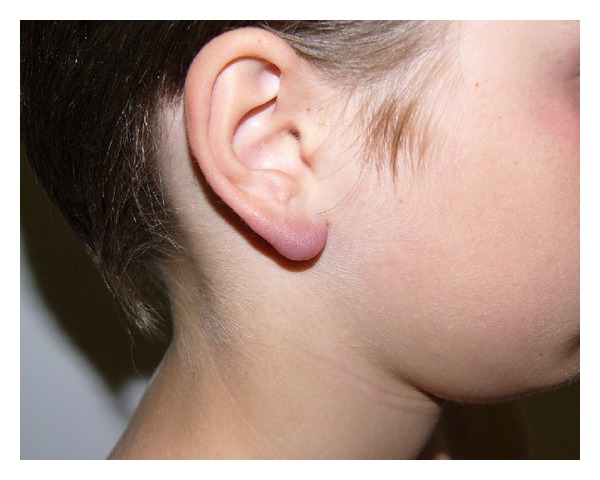
Lymphadenosis benigna cutis.

Data gathered about the other cutaneous features are consistent with those found in literature. LABC is the typical subacute cutaneous manifestation of early disseminated LB (Figure 3). In endemic areas, its frequency is approximately 1-2% and it is the most common kind of B-cell pseudolymphoma of the skin [1].

**Figure 4:**
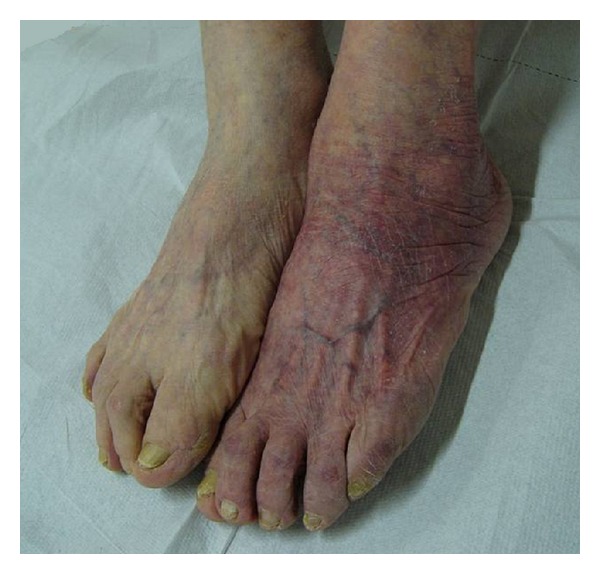
Acrodermatitis chronica atrophicans.

ACA is the typical cutaneous feature of late LB in Europe (Figure 4). It has been described most of all in elderly patients, especially women, and is frequently associated with extra-cutaneous symptoms as reported in other Italian observations [13]. In the United States, only very few cases have been described, referring to imported forms usually [14]. *B. afzelii* has been mostly associated with the development of ACA, even if both *B. garinii* and *Bb sensu stricto* have been isolated from specimens of ACA.

In this survey, we reported 16 patients presenting skin manifestations considered as atypical forms of LB such as lichen sclerosus and atrophicus, morphoea, and anetoderma [1, 15–18]. These manifestations have been more frequently observed in Europe than in the USA although this association remains a controversial topic [15–19].

**Figure 5:**
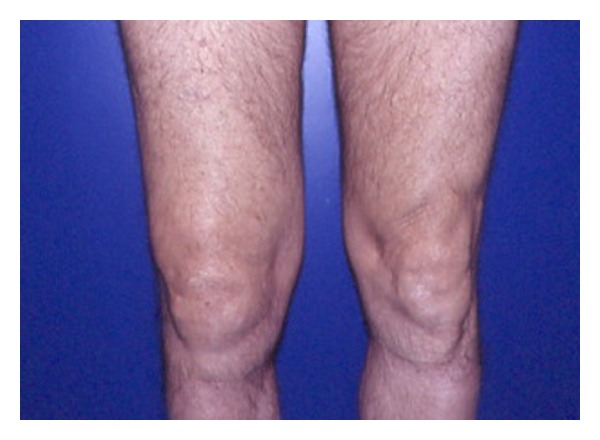
Articular involvement in Lyme borreliosis.

In untreated LB the joint manifestations can arise months to years after the tick bite, usually in the form of a chronic mono or asymmetrical oligoarthritis. In our series the musculoskeletal involvement appears to be frequent. A not very painful arthritis characterized by brief attacks of joint swelling (Figure 5) was observed with an incidence comparable to Northern America data [19]. The percentage of acute arthritis evolved in chronic arthritis recorded in this survey is similar to the one of other endemic areas in Europe [20].

Manifestations of acute peripheral nervous system involvement in LB include cranial neuropathy (peripheral 7th nerve palsy), eventually associated with radiculopathy (“Garin-Boujadoux-Bannwarth” syndrome) or radiculoneuritis (acute onset of severe localised radicular pain and/or motor weakness with or without sensory loss) [21]. In stage 3, irreversible neurological damage is present and the course of the illness is not self-limited. Chronic progressive meningoencephalitis, characterized by spastic paraparesis, cranial neuropathy, or cognitive impairment has been reported in Europe, while Lyme encephalopathy, a mild, late neurologic syndrome manifested primarily by subtle cognitive disturbances, has been reported in the United States [19, 20].

Our data demonstrated that the nervous system has been involved in 65.24% of the analysed population, also presenting severe forms, according to the data recorded in Europe. In Europe, the frequency of neuroborreliosis seems higher, potentially due to the greater neurotropism of *B garinii*, which has not been isolated in North America [3].

There is much controversy about whether *Bb* infection can cause psychiatric disease. In this survey, psychiatric manifestations seem to be quite frequent, but these data are comparable to those recorded in other endemic areas in Europe and can be explained by the high tropism of *Bb sensu lato* for the nervous system, in particular it can cause both a transitory and a permanent damage of the limbus, which is referred to the personal moods and behaviours [3].

Ocular involvement in LB, possible at every stage of the disease, is most frequently reported in European patients in the late phases of the disease [22, 23]. In this study the involvement of the eye has been reported in 10.07% of the patients, especially in women.

As reported in literature, the cardiac manifestations tend to appear one week to seven months after the tick bite (median, 21 days). Borrelial carditis is a relatively rare complication in Europe, occurring in about 1% of all cases (in contrast to 4–10% of North America) [20–24]. In our survey, 8.8% of patients reported symptomatic cardiac involvement associated with *Bb* seropositivity often accompanied by other manifestations, such as EM or neurological deficits. The high number of heart disease found in our series may be connected to the main limitation of our study, the recruitment of seropositive patients that can undoubtedly lead to over-diagnosis of LB. It is well known that the persistence of antibodies is common and it can usually be misconstrued as an evidence of florid infection. It is possible that symptoms such as dizziness, palpitations, or syncope, caused by disturbances of intracardiac impulse generation or impulse conduction and which usually resolve in few weeks, may be considered a coincidental finding in our patients.

Fever was detected in about 25% of the medical records analysed. In Europe the borrelial infection tends to remain localized for a long time without developing severe systemic symptoms and fever is often described during the prodromic phase. Otherwise in Northern America, flu-like symptoms, including fever, may accompany the early cutaneous lesions or can be the first manifestation of the disease. Such differences may be likely caused by the higher capacity of haematic dissemination of *Bb* serotypes found in the New Continent. Data about the prevalence of the fever gathered in this survey are consistent; however, the possibility of coinfections by other bacteria, viruses, and protozoa which can also be transmitted by a tick bite must be taken into account in order to reduce the role of *Bb* in the pathogenesis of the fevers observed [25].

In 5.81% of the patients, laboratory tests showed a high value of liver cyte-necrosis enzymes. Hepatic involvement is not usually a typical characteristic of LB, even if in literature sporadic cases of hepatitis are described [3]. Nevertheless, the alteration of hepatic enzymes in more than 5% of the samples examinated is still a very high record: even in this situation there may be a coinfection transmitted by a tick-bite. It can be assumed that the transaminase increasing can be caused by a coinfection of *Anaplasma phagocytophila*, in which hepatic alterations are more common [26].

This survey provides a detailed picture of the various clinical features described during the different stages of *Bb* infection in seropositive patients living in Friuli Venezia Giulia.

The main limit of the study is that it has been performed on a sample of seropositive patients that could mean a mixture of patients with active *Bb* infection, immunological memory, or nonspecific cross-reactivity. For this reason our criteria of selection were very close: the diagnosis of LB is mainly clinical, therefore clinical criteria (history, symptoms, and signs) resulted decisive for the assignment of the diagnosis and the interpretation of the serological findings. Anyway, in our results this bias must have been taken into account.

## Figures and Tables

**Table 1:** The clinical manifestations observed in the 705 patients seropositive for *Borrelia burgdorferi*.